# Do We Have the Right Performance Indicators for the Circular Economy? Insight into the Swiss Waste Management System

**DOI:** 10.1111/jiec.12506

**Published:** 2016-10-29

**Authors:** Melanie Haupt, Carl Vadenbo, Stefanie Hellweg

**Affiliations:** ETH Zurich–Institute of Environmental Engineering John-von-Neumann-Weg 9, 8093 Zürich, ZH Switzerland

**Keywords:** circular economy, closed-loop, industrial ecology, material flow analysis, municipal solid waste, open-loop

## Abstract

A material flow analysis of the 2012 Swiss waste management system is presented, highlighting the material content available from waste. Half of municipal solid waste (MSW) is materially recycled and the other half thermally treated with energy recovery. A key component of an industrial ecosystem is increasing the resource efficiency through circulating materials. Recycling rates (RRs), an indicator for the circulating behavior of materials, are often used as measure for the degree of circularity of an economy. This study provides an in-depth analysis of the recycling of paper, cardboard, aluminum, tinplate, glass, and polyethylene terephthalate (PET) from MSW in Switzerland by splitting the RRs into closed- and open-loop collection rate (CR) and RRs. Whereas CR refers to collected material that enters the recycling process, RRs measure the available secondary resources produced from recycling processes. For PET, the closed-loop CR of 45% and the open-loop CR of 40% compare to an RR of 31% and 37%, respectively (including exports and recycling of polyethylene and metals from collection). Official collection rates for paper and cardboard are very high (97%), whereas CR of 74% and 89% and RR of 59% and 81% for paper and cardboard, respectively, were found in the present study (including export). For a majority of the separately collected materials investigated, the rates that are determined are substantially lower than those that are officially communicated. Furthermore, given that official rates often do not provide information on the availability of secondary materials, the improvement potential for increased resource recovery is hidden.

**Supplementary Information:**

The online version of this article (doi:10.1111/jiec.12506) contains supplementary material, which is available to authorized users.

## Introduction

Based on the analogy of biological ecosystems, Frosch and Gallopoulos (1989) envisioned an industrial ecosystem characterized by highly optimized consumption of energy and material, minimal waste generation and pollution, and the use of any occurring residues as raw materials for other processes. A key component of the ideal industrial ecosystem is to increase resource efficiency through circulating material. In recent years, the closely related term “circular economy” has gained much attention. The concept of circular economy conceives of a production and consumption system with minimal losses of materials and energy through extensive reuse, recycling, and recovery (Ellen MacArthur Foundation 2013; EEA 2014). The material efficiency and the improvement potential of a society can be quantified through a material flow analysis (MFA). Mapping national waste flows with MFA allows for both monitoring of pathways for material uses and for closing loops of materials (Brunner and Rechberger 2004). For Switzerland, the case study region in this article, several MFAs have been performed on the national waste management system. Liechti (2009) and Dettli and colleagues (2014) focused on the overall material and energetic recovery potential of all waste streams. Steiger (2014) investigated the composition of Swiss municipal solid waste (MSW) and quantified the materials and their energy content contained within. These studies, however, did not model the recycling system on a detailed level, neglecting the ultimate fate of separately collected materials. Therefore, they did not offer any insights to the actual resource conservation and its improvement potential in the Swiss waste management system.

National recycling rates, an often-used measure for the extent of resource efficiency reached within a society, do not share one common definition (e.g., EC 2008, 2015; FOEN 2013a; VKU 2014; EUWID 2012). National statistics often refer to the amount of material collected in relation to the amount in goods consumed (EUWID 2012). This collection rate does not reflect the secondary material produced, but only the input into recycling systems. Within the European Union (EU), the action plan for circular economy (EC 2015) includes the aim of a harmonized definition of recycling rates based on the input into the last recycling step (after sorting out impurities). Rigamonti and colleagues (2012) propose an indicator for material recovery that focuses on the production of secondary materials instead of collection rates. Unfortunately, most studies fail to include the quality of secondary materials and, consequently, do not allow for estimating the displacement resulting from recycling (Geyer et al. 2015). Rates for closed-loop recycling, which implies that secondary material is recycled back into the same product, and open-loop recycling, where the secondary material is used to manufacture something that differs from the preceding product, are often not published individually, but communicated as one rate. This is problematic because open-loop recycling is commonly found in situations where recovered secondary material is used in product systems with lower quality requirements. Improving recycling efficiency for a more circular economy hence includes a transition from open- to closed-loop recycling (Graedel et al. 2011). The environmental benefits of recycling, however, do not necessarily correlate to the distinction of closed- and open-loop recycling, but are mainly dependent on the difference in impacts arising from supplying equivalent products from either primary or secondary material (Geyer et al. 2015; Meylan et al. 2014).

This article presents an MFA of the Swiss waste management system with detailed subsystem MFAs for materially recycled fractions of MSW (paper, cardboard, glass, polyethylene terephthalate [PET] bottles, tinplate, and aluminum). Recycling rates are calculated and disaggregated into open- and closed-loop collection and recycling rates (RRs), showing the contribution of the MSW management system to circular economy and highlighting improvement potentials. This detailed investigation of RRs and inherent material qualities allows one to model replacement rates of primary materials more realistically than has been done in previous studies.

## Methods

### Material Flow Analysis: System Definition and Data Acquisition

**Figure 1:**
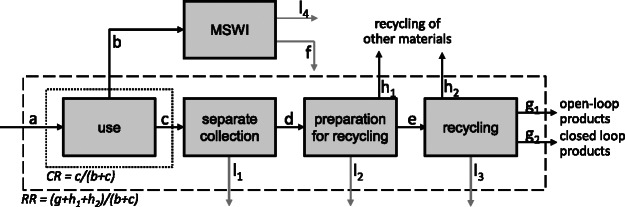
Flows related to a simplified waste management system for separately collected recycling fractions. Boxes indicate the main processes. l_i_ indicates removed impurities and yield losses, whereas f stands for materials recovered from MSWI bottom ash and later delivered to material recycling processes. When material enters the recycling system, it may be recycled for an open- or closed-loop product application (g_1_ and g_2_, respectively), be sorted out as impurity or lost (l_1_ to l_3_), or it is sorted out as a different material, but still enters a recycling process (h_1_ and h_2_, e.g., polyethylene caps in the polyethylene terephthalate bottle recycling). Figure adapted from UNEP (2011). The dotted line indicates the system boundaries for the collection rate (CR), and the dashed line shows the system boundaries of the recycling rate (RR). MSWI = municipal solid waste incineration.

**Figure 2:**
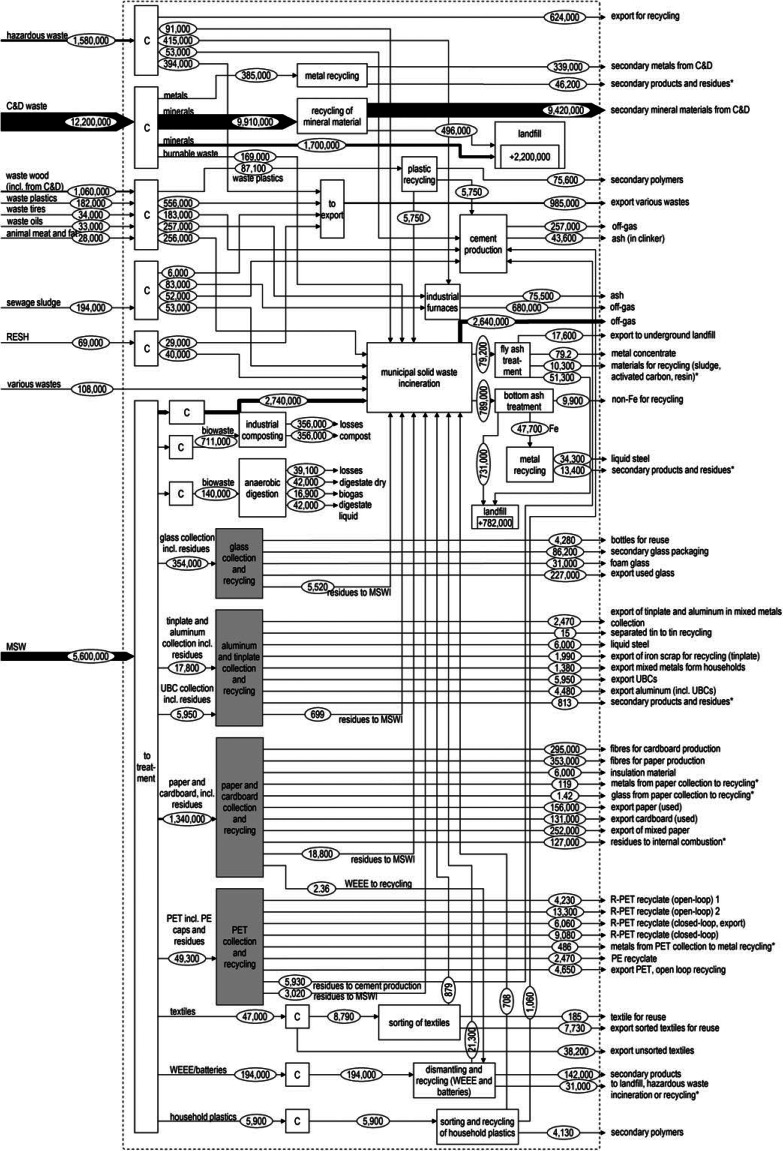
Material flow analysis of the Swiss waste management system in 2012 in tonnes per year. Gray boxes mark subsystems for which the detailed MFAs are shown in figures 3 to 6. The thickness of the lines indicates the relative magnitude of the flows. The dotted line marks the system boundary of the system (including waste management processes within Switzerland; output of the system are useful secondary materials and exports of material fractions to other countries). Data sources for the values can be found in the supporting information S1 on the Web. (*): exports where composition or final destination is unknown, to export: no physical process, aggregation of export flows for illustration purpose, off-gas: amount of waste transferred to emissions during thermal process; C = collection; RESH = residues from shredder; UBC = used beverage cans; R-PET = PET from recycled material; C&D waste = construction and demolition waste; MSW = municipal solid waste; BA = bottom ash. PE = polyethylene; PET = polyethylene terephthalate; WEEE = waste electric and electronic equipment.

The MFA of the 2012 Swiss waste management system is based on data presented in Dettli and colleagues (2014) for overall waste generation, and includes quantities treated in waste incinerators and industrial furnaces, in cement production, and amounts exported. The geographical scope encompasses the waste streams occurring in Switzerland, but the import of waste is excluded, because the treatment therefrom is not part of the required functionality of the Swiss waste management system. The MSW includes all wastes from households or similar waste from small industries (including the recycling fractions from these sources).The construction and demolition (C&D) waste includes the metals and minerals as well as burnable waste from the building sector (except for unpolluted excavated soils). Hazardous waste (including hazardous waste from households) is based on statistics from the Federal Office of the Environment (FOEN) (FOEN 2013b), with the main contributors being chemical wastes, polluted soil, and treatment residues and slurries. Industrial wastes that are directly used within the plant for energy recovery (e.g., waste at paper mills) or that are used as raw materials in other processes (e.g., waste from saw mills used in board production (FOEN 2013c) (or locally used agricultural wastes) are omitted. Compared to Dettli and colleagues (2014), adaptations are made regarding MSW to incineration: Impurities in separately collected fractions are included in the waste streams for recycling (flow a in figure 1) to enable for an analysis of the quality of the separate collection systems. As an output of the recycling activities, these residues are then sent to MSW incineration (MSWI) (figure 2). Metals ending up in MSWI are increasingly recovered in bottom ash treatment plants, and these fractions are added to the work of Dettli and colleagues (2014) based on Hügi (2012).

### Determining Recycling Rates

**Figure 3:**
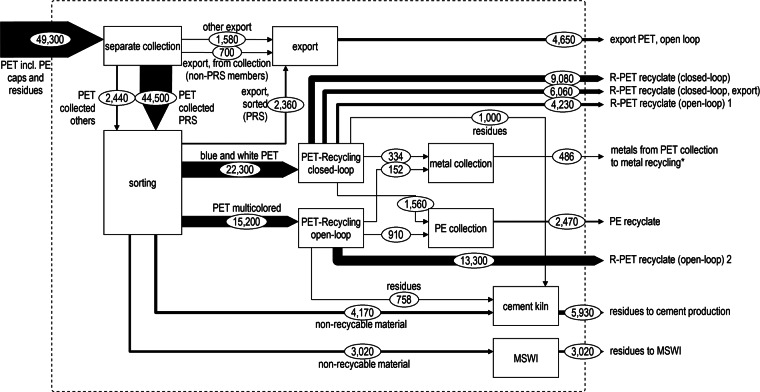
MFA of PET bottle recycling in Switzerland 2012. Input is the material collected for recycling including all contaminants. All materials produced and flows into further treatment processes (e.g., MSWI) are shown as exports. Flows are given in tonnes per year. The thickness of the lines indicates the relative magnitude of the flows. Data sources for the values can be found in supporting information S1 on the Web. MFA = material flow analysis; MSWI = municipal solid waste incineration; PE = polyethylene; PET = polyethylene terephthalate; PRS = association PET Recycling Switzerland; R-PET = PET from recycled material.

**Figure 5:**
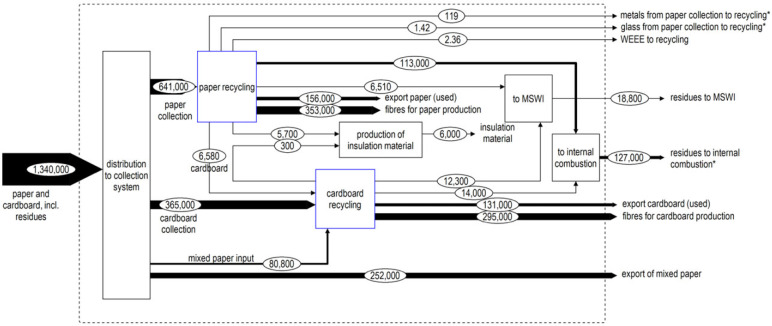
MFA of paper and cardboard recycling in Switzerland 2012, following the same format as figure 3. MFA = material flow analysis; MSWI = municipal solid waste incineration; WEEE = waste electric and electronic equipment.

**Figure 6:**
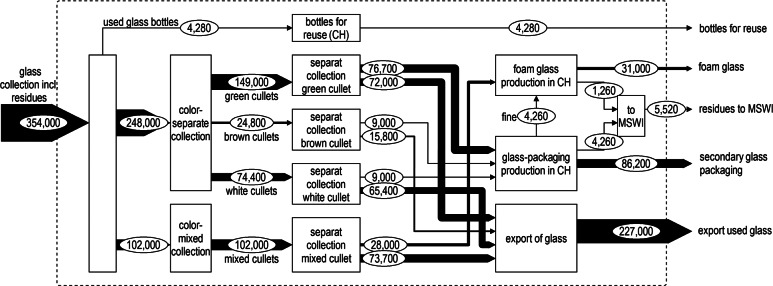
MFA of glass recycling in Switzerland in 2012, following the same format as figure 3. CH = Switzerland; MFA = material flow analysis; MSWI = municipal solid waste incineration.

**Figure 7:**
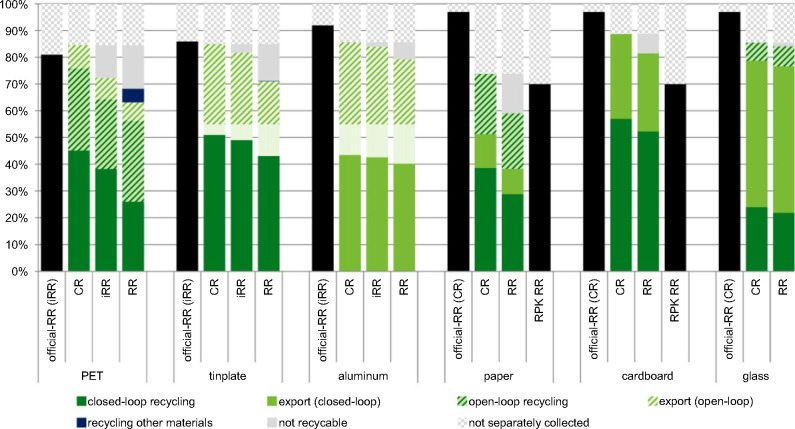
Recycling rates of the Federal Office for the Environment (FOEN) compared to closed- and open-loop recycling and export rates found in this study. Material marked as “not separately collected” is sent to MSWI from the households; “not recyclable” denotes material that is sent to MSWI or cement kilns after being collected separately. Materials recovered from MSWI bottom ash are omitted because they do not appear in official-RRs. Official-RR = recycling rates of the FOEN (description can be found in table S1-2 in the supporting information on the Web), CR = collection rates; iRR = intermediate recycling rates; MSWI = municipal solid waste incineration; RR = recycling rates.

In addition, in-depth MFAs of the MSW recycling systems of paper, cardboard, PET bottles, glass, tinplate, and aluminum are performed. Data are collected from the recycling industry and related associations, as well as from national statistics. All data sources are given in supporting information S1 available on the Journal's website. The consistency of the available data varied significantly between waste streams, and data reconciliation, mainly consisting of additional data mining, is therefore carried out together with the respective industry and the Swiss FOEN. On the basis of the aforementioned in-depth analysis of recycling processes, the official recycling rates (official-RR) communicated by FOEN (FOEN 2013d) are recalculated and disaggregated into domestic closed- and open-loop collection and recycling rates, as well as export rates. A simplified scheme of the MFAs of the recycling systems is presented in figure 1, illustrating the difference between the separate collection rate (CR) and the RR (adapted from UNEP 2011). The structure of all in-depth MFAs (figures 3 to 6) is based on this simplified scheme. As can be seen in figure 1, the CR describes the ratio between the amount measured in separate material collection (flow c) and the overall quantity of waste generated within the respective time frame (sum of flows b and c). The RR is given as the ratio between recycled materials (flows g_1_, g_2_, h_1_, and h_2_) and waste generated (flows b+c). When the overall RR is split into an open- and closed-loop RR, the recycling of other material (flow h_1_ and h_2_) is shown as a separate rate and is consequentially not included in the open- or closed-loop RR. In Switzerland, the official RR for some materials are CR, whereas for other fractions intermediate RRs (iRRs) are used (ratios d/(b+c) in figure 1, below called iRR). Details on the definition of official-RR can be found in figure 7 and in supporting information S1 on the Web. Mass flows shown in figures 3 to 6 include impurities and other materials and do not only refer to the investigated recyclables. Impurities from the use phase and collection are included in flows b, c, d, e, and f. Gray arrows indicate removed impurities and yield losses along the process chain. Exports, denoted as CR-export or RR-export, can occur either after separate collection or after recycling, respectively, as a ready-to-use secondary material.

**Figure:**
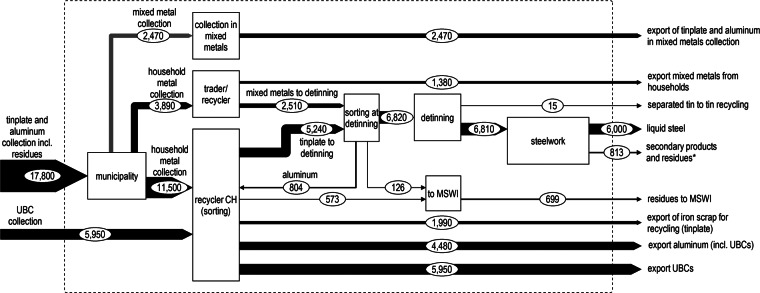
MFA of aluminum and tinplate recycling in Switzerland 2012, following the same format as figure 3. CH = Switzerland; MFA = material flow analysis; MSWI = municipal solid waste incineration; UBC = used beverage cans.

Recycling processes that allow for the return of the recycled material to the preceding application are classified as closed-loop recycling. Open-loop recycling describes the preparation of a secondary material that cannot be used in the same product as in the previous service life (EU-JRC 24916 EN 2011; ISO 14044 2006; EU-JRC 24708 EN 2010, 346 ff.). The terms “closed-loop” and “open-loop” are therefore here used to describe the opportunity space of the material's usage, meaning the possible applications of the materials in their next life cycle. All specific assumptions are listed in table 1. Wherever possible, the ultimate fate of exported material (closed-loop, open-loop) is indicated.

**Table 1:** Definition of closed-loop and open-loop recycling pathways for the investigated material fractions

	Treatments in Switzerland		
Material	Closed-loop recycling	Open-loop recycling	Treatments abroad
PET	Production of bottle-grade PET	Manufacturing of PET to be used in PET packaging or PET fiber production	Open-loop recycling: manufacturing of PET for packaging or PET fibers (Würmli 2012)
Tinplate	Remelting of detinned tinplate cans	Remelting of steel scrap without previous detinning	Open-loop recycling, because no detinning takes place
Aluminum	(No recycling plant based on postconsumer scrap available in Switzerland)		Remelting of used beverage cans is assumed to be closed-loop; remelting of mixed aluminum fractions as open-loop; and pathways remain unknown.
Glass	Bottle reuse and production of packaging glass (bottles or jars)	Production of foam glass or glass sand	Closed-loop assumed, given that abroad also mixed colored waste glass are sorted by optic detectors (Städler 2012).
Paper	Preparation of fibers for paper production	Use of fibers in cardboard production or as insulation material	Assumption closed-loop; fate remains unknown.
Cardboard	Production of fibers for cardboard production	Use of fibers as insulation material	Assumption open-loop; fate remains unknown.

*Note*: An expert estimate or “best-guess” assumption is provided for the fate of each exported material.

PET = polyethylene terephthalate.

## Results

The total waste produced in Switzerland in 2012 amounts to 21 million metric tonnes or approximately 3 tonnes per capita per year (figure 2). C&D waste, at over 13 million tonnes (Mt), is the most important waste stream in terms of mass (58% of total). Most of it is recycled, for example, as concrete granulate and other mineral filler (Dettli et al. 2014). Separately collected fractions other than MSW (waste wood, waste plastics, waste tires, etc.) accounted for 6% and hazardous waste for 7% of the total waste occurring in Switzerland. A substantial share of both the separately collected fractions (42%) and the hazardous waste (65%) is exported for treatment. MSWI plants treated 48% of MSW (2.6 Mt), whereas the CR for recyclables from MSW was 52% (2.9 Mt). In the subsequent recycling processes, 6% of the input material was identified as impurities or otherwise rejected and disposed of in MSWI, cement kilns, or on-site furnaces at paper and cardboard production facilities. For this reason, the fraction of Swiss MSW that can be assumed to be recovered is reduced to 50% (2.7 Mt), of which 27% is exported for recycling. Figure 2 shows the complete MFA of the Swiss waste management system.

The material-specific subsystems of MSW for PET, paper, cardboard, glass, aluminum, and tinplate (marked as gray boxes in figure 2) are presented next. The MFAs for paper and cardboard as well as aluminum and tinplate are shown as combined MFAs given that these material cycles are heavily interlinked, particularly through mixed collection systems.

### Polyethylene Terephthalate Bottles

Figure 3 shows the detailed MFA of the PET recycling system and processes split into a separate collection (mostly taking place at retailers), a sorting step that normally takes place in one of five sorting centers within Switzerland, and the recycling processes. After color sorting, the blue and colorless material fractions can be used in closed-loop recycling processes because of a caustic soda washing process that allows for bottle production from 100% recycled material (Lemann 2005; RecyPET AG 2012). PET fractions of other colors are sent to open-loop recycling for use as PET fibers and food packaging, such as cookie trays. These fractions cannot be used for bottle production because the color hampers the production of bottles attributed to design restrictions regarding visual appearance. During both recycling pathways, polyethylene (caps) and metals (impurities in the collection) are sorted out and sent to the respective recycling process for which closed-loop recycling was assumed. Residues from sorting are treated in either MSWI plants or cement kilns, whereas waste occurring during the recycling process is only used as alternative fuel in cement kilns. A fraction of closed-loop recycled secondary bottle-grade PET is exported. This finalized product, however, is included in the closed-loop RR instead of the export rate because the recycling process takes place in Switzerland. The CR for closed-loop recycling of PET is 45%, but only 26% of the PET waste generated is later available as granulate for further bottle production (closed-loop RR). Fines, 20% of the input into closed-loop recycling (figure 3), are sorted out and enter the open-loop recycling system, diminishing the closed-loop RR compared to the CR and keeping the open-loop rate constant, even though residues are sorted out. The aggregated CR (which includes material for open- and closed-loop systems as well as export) found in this study reaches 85%, whereas the official-RR is 81% (derived analogously to iRR). The iRR is also calculated for PET and found to be 72%. The reason for the difference between the official-RR and the iRR can be explained by the assessment of impurities. In the official-RR, a fixed percentage of the collected material is deducted as an assumed fraction of impurities according to FOEN (2013a), whereas in this study the actual flows of 2012 were used. Finally, 9% of the PET collected (including impurities) was exported to other countries, where open-loop recycling is expected (Würmli 2012).

### Aluminum and Tinplate

Aluminum and tinplate from MSW are increasingly collected as a mixed metal fraction in a common container to facilitate logistics and reduce transportation cost. The shares of metal collected as mixed metal fraction and as single metal fraction (as aluminum or ferrous metals) from households are, however, unknown, given that data on the collection system are not gathered from the recyclers or traders. The municipalities or local traders collect the material from collection points, whereupon it is delivered to recyclers inside or outside of Switzerland. The mixed material is sorted using magnetic and eddy current separators. Because no aluminum smelting plant exists in Switzerland, aluminum scrap is exported for recycling (mainly to Germany and Italy, with smaller shares going to Austria and France) (Swiss-Impex 2012). Ferrous scrap is partly detinned in Switzerland (60% of collection) and then used in Swiss steel works. The other 40% of the collected ferrous scrap are exported for remelting without previous detinning. Scrap from detinned cans is used for steel production in electric arc furnaces in Switzerland. From this scrap, liquid steel with many possible applications is produced. If the material is not detinned, a dilution of tin with higher-quality scrap is necessary in the concurrent recycling process, leading to dilution losses (Verhoef et al. 2008; Nakamura et al. 2012; Ohno et al. 2014). For this reason, only melting of detinned material is counted as closed-loop recycling. The amount of tinplate in MSWI is largely unknown and was assumed to be 2,084 tonnes per year based on the official-RR, although the collected amount, calculated based on internal reports of the related recycling association, differs from the official statistics. In comparison, Steiger (2014) estimates, based on a sampling campaign, that 19,000 tonnes of ferrous metals are delivered to MSWI, of which 9,000 tonnes (including multiple fractions) may be realistically recycled. But this estimate includes large uncertainties (values in the range of 0.7 to 7.0 kilograms per year and person were found for the 33 investigated municipalities) and was therefore not used. Whereas the assumption of 2,084 tonnes in MSWI leads to a recycling rate of 71% (official-RR, 86%), an amount of 9,000 tonnes in MSWI would lead to a CR of 43%.

Metals are frequently regarded as materials that can be infinitely recycled (Verhoef et al. 2008), but because of a tolerance for higher levels of impurities, aluminum postconsumer scrap is mostly used for castings in automotive applications (Gesing and Wolanski 2001; Das et al. 2007; Modaresi and Müller 2012; Reck and Graedel 2012), and therefore its recycling occurs mostly in open-loop fashion (Müller and Løvik 2015). Wrought alloys, the major product of the primary aluminum industry, require an alloying elements content below 10% and can therefore not be produced from old scrap (Allegrini et al. 2015). Concentrations of impurities (alloying elements from the material's previous use or from collection), are lowered during remelting through dilution with primary aluminum or higher-quality scrap (Løvik and Muller 2014). The separate collection of used beverage cans (UBCs) is of great importance for the recycling of aluminum, given that this source separation according to use also leads to a sorting by alloys. Separately collected UBCs can be melted to produce new beverage cans, but a mixed scrap fraction will be used for different products (Müller and Løvik 2015; da Silva et al. 2010). The official-RR only considers separate collection of UBCs, but the CR and RR below also include aluminum packaging. In 2012, the share of aluminum from MSW collected separately as UBCs amounted to 43% (closed-loop CR-export). Of the aluminum, 42% was source sorted as mixed fraction (CR-export, partly mixed with other metals). Treatments abroad were not modeled in detail, but it was assumed that material exported included 5% impurities (as quantified for the mixed collection in Switzerland) that would occur as losses in the melting process. To account for the yield losses and impurities removed during the melting of ferrous scrap, efficiencies (88% yield) were based on Remus and colleagues (2013). For aluminum, an efficiency of 94.4% was used based on a study on UBCs (da Silva et al. 2010).

Materials recovered from MSWI are not included in recycling rates, but also contribute to circular economy. Assuming a recovery rate of 80% for ferrous scrap from MSWI bottom ash (Bunge 2009) (85% found in Denmark [Allegrini et al. 2015]), the iRR for tinplate would increase by 12% and the RR by 9%, resulting in an RR of 80%. For aluminum, Allegrini and colleagues (2014) found an average recovery efficiency of 62% from bottom ash. Assuming a recycling efficiency of 70% (based on Allegrini et al. [2015]), the iRR would increase by 9%, whereas the RR increases by 6%. This results in an overall RR of 85% for aluminum.

### Paper and Cardboard

Paper and cardboard are collected from households either as mixed or separated materials. The collection system differs for each municipality. Mixed material is either sorted by recyclers or paper recycling industries or used in the cardboard production. The share of material that is collected mixed, but separated in an intermediate step, is largely unknown, because the data were gathered at recycling industries, not at the municipal level. Therefore, only material arriving as mixed at the recycling industries, which is a minor fraction of the mixed collection, can be shown in the MFA (figure 5). The collected cardboard includes various qualities, such as folding boxboard, chipboard, and old corrugated containers. Given that cardboard hampers the paper recycling process, it is assumed that recyclers ensure that no cardboard from the separation process ends up in paper recycling (a maximum 2% is allowed) (DIN EN 643 2014). By contrast, paper can be used in the cardboard recycling process. According to industrial experts, the sorted cardboard fraction still contains roughly 7% of postconsumer paper attributed to imperfect sorting, increasing the open-loop RR of 17% (paper directly used as input in cardboard industry) to 23% for paper. As mentioned above, data were collected from the paper industry, who often buys presorted material. It is therefore unknown how much paper is collected as mixed with cardboard and separated at a recycler. For this reason, the downcycling of paper is underestimated.

Twenty percent of the total amount collected is not recyclable. This material, mostly consisting of fibers too short for reuse, is used for heat production in internal furnaces of the paper industry. Thirteen percent of paper and 32% of cardboard collected for recycling are exported. It was assumed that this material is recycled in closed-loop recycling processes.

The CR found in this project is substantially lower than the official-RR for 2012. The models for the calculation of the official-RR (given as CR) of the Swiss association for paper and cardboard recycling (Recycling Papier und Karton; RPK), however, have since been revised in collaboration with the authors of this article. Adapted recycling rates were communicated starting from 2015, but corrected values are available starting from 2012. The corrected value for 2012 from RPK is shown for comparison in figure 7. Both the presented numbers as well as the newly defined CR of RPK include nonrecyclable toilet and kitchen tissues, therefore substantially lowering CR rates.

### Glass

Glass collected in the municipalities is either color-separated (70%) or, to facilitate logistics, collected as mixed-colored waste glass (29%). The collection not only includes glass bottles, but also packaging glass as jars. The remaining 1% of the collected glass is for reuse (returnable bottles). Although mixed-color cullet is used to produce foam glass in Switzerland, any exported waste glass is sorted by color and used to produce packaging glass; therefore, it can be assumed that exported material enters closed-loop recycling. The closed-loop CR is 24% (excluding exports), of which 92% are available for secondary glass production. Four percent are collection residues and 4% is fine material that is later downgraded and used in open-loop recycling processes. The open-loop RR for Switzerland is 7%. The total RR found here is substantially lower than officially communicated (84% vs. 97%, respectively). The large difference occurs because different statistics are used for the glass entering MSWI (flow b in figure 1): The FOEN estimates the amount of glass bottles consumed and extrapolates to all packaging glass, whereas this study is based on the composition of residual waste in Steiger (2014). Therefore, 60,000 tonnes of glass in residual waste were included in the MFA (Steiger 2014), although, according to the calculation using the official rate and returned amounts based on the FOEN (2013b), only 14,000 tonnes were expected. First estimates of the mass of glass packaging imported by private shopping abroad showed that an amount of 16,000 tonnes can be expected to be imported into Switzerland (Wullschleger 2012). This amount has, so far, not been considered in the official statistics, partly explaining the deviations. In addition, the estimations of the amount of waste glass not stemming from glass bottles, but other glass packaging, include large uncertainties.

### Comparison of Recycling Rates

From these detailed material-specific MFAs, we can now deduce open- and closed-loop RR and RR-export as given in figure 7. The calculations can be found in supporting information S2 on the Web. The official-RR (CR or iRR, as given in figure 7) of 2012 are taken from FOEN (2013b). For PET, a closed-loop CR of 45%, an open-loop CR of 31%, and a CR-export of 9% are found. The corresponding RRs are 26%, 30%, and 7%, respectively. Within open-loop recycling, the rate increases from CR to RR attributed to inputs of low-quality material from the closed-loop collection. For paper and cardboard, the RRs found are substantially lower than communicated by the FOEN: For paper, total RR and CR amounted to 59% and 74%, respectively, and for cardboard 81% and 89%, respectively. The official-RR was 97% for paper and cardboard. The RPK's calculation model, which is the basis for the rates published by FOEN, has since, however, been adapted in collaboration with the authors of the present study, and the official-RR has been changed to 70% in 2014 (combining the CR and CR-export of cardboard and paper, also added in figure 7) (RPK 2012).

## Discussion

### Swiss Waste Management System

The generation of MSW in Switzerland is one of the highest in the world at 700 kilograms per person per year. In 2012 (and presently), around 50% of the waste undergoes incineration, whereas the other half enters material recycling. Within the material recycled fractions from MSW, paper and cardboard is, by far, the biggest fraction, with 47% (mass-wise), followed by 30% biogenic waste, 12% glass packaging, 7% waste electronic and electric equipment (WEEE), 2% PET and waste plastics other than PET, 2% textiles, and 1% metals (aluminum and tinplate). Not analyzed in detail is the recycling of WEEE (attributed to the complexity of the system), textiles (poor data availability), and waste plastics (currently in a transition period; a large-scale recycling system has only been set up for household plastics in 2012).

### Recycling Rates

The official rates communicated by the Swiss FOEN are often collection rates or intermediate recycling rates (including, e.g., the removal of impurities, but omitting recycling yields). Within the EU, mostly CRs are currently used, but a change to iRR is foreseen and the application of RR has been discussed (EUWID 2012; EC 2015). Only RR describes the amount of material ultimately recovered from the waste management system. For this reason, RRs should be used as performance indicators for the circular economy to ensure that the efficiency of the recycling processes is considered. Accounting for the recycling yields, however, creates a need to carefully define individual RR targets for each material, accounting for technical feasibility. Today, neither the Swiss nor the European rates give information about the secondary material that is actually recovered. Closed- and open-loop RRs should, however, be reported separately, given that they conform to different resource recovery strategies. Open-loop recycling often enables the use of a wide range of material qualities and therefore maximizes the amount of waste recycled. In turn, closed-loop recycling targets also emphasize quality aspects, so that the material serves in the same product spectrum as the original good. In closed-loop recycling, higher flexibility is maintained in terms of recycling and treatment options after the use of the material, but open-loop is irreversible, indicating that the respective materials are degraded in quality and cannot again be used in the original application, therefore restricting subsequent recycling options. No general statement can, however, be made about the comparative environmental performance of open- and closed-loop recycling, which mainly depends on the substituted materials or products and hence the specific case (Geyer et al. 2015).

For the majority of materials investigated, the RR is substantially below the official-RR communicated. This was analyzed for the case of Switzerland, but is expected to be similar in other countries. Current recycling rates not only struggle to provide an indication on how much secondary material becomes available, but they also hide improvement potentials in waste management. Political decisions based on CR are hence made with potentially insufficient system information. Because of the inconsistent definition of national recycling rates, current rates are not comparable. For this reason, the European action plan for circular economy calls for harmonized recycling rates (EC 2015). A harmonized definition on a global scale would enable the comparison of recycling rates and the identification of global improvement potentials for material recovery.

The amount of material going to MSWI is often assessed with great uncertainty. For example, according to the official-RR, 14,000 tonnes of glass are delivered to MSWI in Switzerland, but Steiger (2014) finds that 60,000 tonnes of glass in the MSW is sent to MSWI from households. An improved monitoring of the material flows to MSWI would enable a calculation of CR and RR with higher accuracy.

Of the source-separated material collected and recovered, 73% is recycled within Switzerland. Recycling of material taking place abroad is also included in the study, but the knowledge of the treatment processes and the material use in open- or closed-loop recycling is limited. In discussion with industry, however, probable treatments were defined (see table 1) and modeled within the MFA, given that the inclusion of exported fractions is of great importance to capture the full consequences and benefits of the waste generated in Switzerland.

### Product Design in Circular Economy

The detailed material-specific MFAs in this study also highlight the influence of product design choices: The amount of PET used in a closed-loop recycling process for bottle production is not only limited by the collection or sorting technology, but also by color, given that only blue and colorless PET bottles are suitable for bottle-to-bottle recycling. The use of green PET bottles as a design choice implies a limited number of recycling options, given that it can only be used in either nonbeverage packaging material or to produce fibers and is therefore excluded from closed-loop recycling processes. In addition to design choices and the sorting ability of consumers, the quality of the recyclables influences their ultimate fate. PET that is collected together with plastic packaging or hollow bodies (like bottles from shampoo or washing detergents, etc.) from various types of plastic is often not recycled in closed-loop recycling (high contamination in collection), but instead used as either alternative fuel in cement kilns (in a mixed residual fraction) or in open-loop recycling. Increasing the recycling rates might come at the expense of poorer quality of separation and thus collected material. This limits the secondary use of separately collected materials and increases the preparation effort needed in recycling processes (e.g., sorting, balling, shredding).

### Recycling Rate as Indicator in Circular Economy

The circular economy action plan of the European Commission (EC) (EC 2015) includes targets for the recycling and reuse of materials to increase the waste management's contribution to circular economy. For a comparison among states or regions, however, a harmonized indicator is needed. For this, defining the recycling rates based on the input into the last production step is discussed (EC 2015). For some materials, this would make the European rate identical to the iRR shown above. It misses, however, incentives to increase the efficiency of recycling. For example, a reduction of the mineral content of ferrous scrap from MSWI bottom ash would not be favorable, given that a lower content of minerals would decrease the iRR. RRs are instead proposed as a measure and as an indicator for comparison between countries, given that they include the recycling process and therefore give incentive to optimize all processes. In addition, they provide information about secondary materials produced from waste.

The question remains whether the primary goal of the circular economy is the supply of alternative resources from secondary sources or the minimization of environmental impact. In the former case, RR is a good performance indicator. The distinction between open- and closed-loop recycling is useful to quantify how much material is truly “circulated,” although the classification into closed- and open-loop recycling should not be expected to correlate to the net environmental benefit (Geyer et al. 2015). If the lowering of environmental impacts is the primary goal, detailed assessments of the various recycling processes are needed, including the modeling of substitution and the associated displacement effects (Zink et al. 2015; Geyer et al. 2015). This is more data intensive than a pure MFA, but gives a broader perspective and therefore is key for an environmentally optimal waste management system. Today's metrics often focus only on the closing of material cycles, with the environmental benefits and impacts often remaining unaddressed. The Waste Framework Directive of the European Union requests, however, the application of life cycle assessment (LCA) to identify cases in which it is reasonable to deviate from the classical waste hierarchy (avoid, reuse, recycle, recover, and landfill) (EC 2008). To provide guidance for future policy making and to develop optimal waste management strategies, a holistic view of the relevant environmental impacts is needed. Therefore, future work is aimed at a full LCA of the Swiss MSW management system. Given that the environmental benefits depend largely on the material substituted by the secondary material and the associated displaced product systems, the MFA presented in this study, especially with regard to the detailed investigation of the pathways in recycling, will be used as a basis. The comparison of LCA results and RR may also reveal potential trade-offs between the goals of resource recovery and lowering environmental impacts, for example, when an increase in recycling effort increases overproportionally attributed to the inclusion of low-quality material for recycling.

### Thermal Treatment in Circular Economy

Material recycling from MSWI bottom ash (figure 2) has, so far, not been included in recycling rates. Material recovery from bottom ash is, however, a viable strategy when moving toward a circular economy (Haupt et al. 2016). Because it is not possible to source separate all materials, especially in the case of increasingly complex product designs, the need for thermal treatment resulting in various residual fractions will most probably remain. The thermal recovery of the energy contained with subsequent material recovery seems a promising concept, as long as the quality of the recovered materials allows for their use in subsequent processes. Given that the material recovery from MSWI contributes substantially to metal recycling, these material flows need to be taken into account when measuring the degree of circularity reached within a society. Whether the MSWI material recovery should be included in the national recycling rate of source-separate material or given as an individual thermal recycling rate needs to be discussed.

Taking material quality into account is key when moving from a linear to a circular economy. Kral and colleagues (2013) highlighted that detrimental substances need to be removed during recycling to facilitate sustained closed-loop recycling of materials. When intrinsic material quality is lower after every use cycle, for example, attributed to higher contents of alloying elements in aluminum or steel, closed-loop recycling will not be feasible in a longer time frame. In addition, previous studies showed that low-quality scrap in metal recycling can lead to an increased electricity demand (Haupt et al. 2016), highlighting the trade-off between material recycling and energy efficiency.

## Conclusions

This study concludes that the currently used rates are not suitable as a performance indicator for a circular economy. Often, collection rates are communicated, but give neither an adequate picture of the available quantity of secondary resources produced nor information about the final destination of these materials. iRRs, as published for some recyclables and in discussion in the European Union, refer to the input into the recycling process, therefore excluding the impurities from collection. iRRs still lack, however, an incentive to improve recycling efficiencies or reduce contaminants of the recyclable good. Therefore, they fail to describe how much material is kept within material cycles. Communicating not only CR or iRR, but also closed- and open-loop RR provides a better starting point to assess the contribution of integrated waste management to a circular economy.

## Supplementary Information

**Supporting Information S1**: This supporting information contains (1) data sources for all wastes streams modeled for the national material flow analysis in addition to data taken from Dettli and colleagues (2014), (2) an overview of the definition of the Swiss recycling rates as defined by the Swiss Federal Office of the Environment, and (3) all data sources used in the material flow analyses of polyethylene terephthalate, glass, aluminum, and tinplate as well as paper and cardboard. **Supporting Information S2**: This supporting information contains a table (table S2-1) that shows the details of the calculations of the recycling rates. Mass flows are taken from the material flow analysis (MFA) in the main article. All sources for the input data of the MFA can be found in supporting information S1.

Supporting info item
