# Supplementary material for: Do We Have the Right Performance Indicators for the Circular Economy? Insight into the Swiss Waste Management System
Source: J Ind Ecol. 2016 Oct 29;21(3):615–27. doi: 10.1111/jiec.12506 (PMC13068685; doi:10.1111/jiec.12506)
Supplement: Supplementary file 1 — Supporting info item [file 44498_2017_2103015_MOESM1_ESM.zip › jiec12506-sup-0001-tables.pdf]

SUPPORTING INFORMATION FOR:

Haupt, M., C. Vadenbo, and S. Hellweg. 2016. Do we have the right performance indicators for the circular economy? – Insight into the Swiss waste management system. *Journal of Industrial Ecology.*

Summary

This supplementary material S1 contains (i) data sources for all wastes streams modelled for the national material flow analysis in addition to data taken from Dettli et al. (2014), (ii) an overview of the definition of the Swiss recycling rates as defined by the Swiss Federal Office of the Environment, and (iii) all data sources used in the material flow analyses of PET, glass, aluminum and tinplate as well as paper and cardboard.

Table of Contents

1. Data sources of material flow analysis Switzerland S1-2
2. Description of officially communicated recycling rates S1-4
3. Data sources for in-depth material flow analyses S1-5
   3.1 PET bottles S1-5
   3.2 Aluminum and tinplate S1-6
   3.3 Paper and cardboard S1-7
   3.4 Glass S1-8

1. Data sources of material flow analysis Switzerland

The comprehensive material flow analysis (MFA) of Switzerland was based mainly on data from Dettli et al. (2014). In addition to Dettli et al. (2014), the MFA includes details about the recycling processes for several wastes. While for PET, glass, aluminum, tinplate, paper and cardboard from municipal solid waste (MSW) an in-depth study was performed, the recycling of construction and demolition waste, waste plastics, waste electric and electronic equipment (WEEE) and batteries was only modelled based on available data. Table S1-1 gives all the sources used for these recycling processes. Values from Guerra and Kast (2015) for construction and demolition waste were available for 2005 and 2015 and a linear interpolation took place to calculate the values for 2012 which are used in the MFA for 2012 (Figure 2 in main article).

Table S1-1: Data sources for construction and demolition (C&D) waste flows, the recycling of plastic from industrial and municipal solid waste, recycling of waste electric and electronic equipment (WEEE) and battery recycling. Reference year for C&D waste as well as plastic recycling is 2012, for battery recycling 2011, for WEEE recycling 2009.

flow	value	unit	source
C&D waste in 2012	12,169,000	t	Dettli et al. (2014)
metals in C&D waste	385,057	t	Guerra and Kast (2015); linear interpolation between amount in 2005 and 2015
C&D waste to MSWI	169,000	t	Dettli et al. (2014) (Guerra and Kast (2015) slightly higher (7%))
direct landfilling	1,700,000	%	Dettli et al. (2014)
efficiency of minerals recycling	95	%	Schneider and Rubli (2007): 5% losses in preparation for recycling; for recycling, 100% efficiency is assumed as mineral material is mostly only grinded or crushed
efficiency of metal recycling (from C&D waste)	88	%	Based on efficiency of steel production (Remus et al. 2013)
plastic from industry, recycling yield	86.8	%	Schelker and Geisselhardt (2011)
plastic from industry, recycling residues	50% MSWI, 50%cement industry		Schelker and Geisselhardt (2011)
plastic from households, recycling yield	70	%	Tschümperlin et al. (2015)
plastic from households, recycling residues	40% MSWI, 60%cement industry		Tschümperlin et al. (2015)
WEEE recycling	all transfer coefficients		Wäger et al. (2011)
yield metal recycling from WEEE	88	%	value for ferrous metals from Remus et al. (2013) assumed for all metals
battery recycling (from household or lead batteries)	all transfer coefficients		Batrec Industrie AG (2012); same values assumed for all batteries based on mass balance from Batrec (reference year for transfer coefficients 2011)
textile recycling	all values		Meier (2015)

Table S1-2: Data sources for the modelling of municipal solid waste incineration (MSWI), treatment of bottom ash including metal recycling and treatment of fly ash.

flow	value	unit	source
outputs of MSWI	all transfer coefficients		internal document of Federal Office of the Environment (residues from thermal treatment 2012)
fly ash to underground landfill	22	%	internal document of Federal Office of the Environment (residues from thermal treatment 2012)
fly ash to acidic fly ash leaching / FLUREC	43	%	internal document of Federal Office of the Environment (residues from thermal treatment 2012); 20% are treated in FLUREC process
fly ash to filter cake in acidic fly ash leaching	70	%	Boesch et al. (2013)
fly ash to solidification/landfill	34	%	internal document of Federal Office of the Environment (residues from thermal treatment 2012)
FLUREC process yields	all values		annual report KEBAG (operator of FLUREC process, KEBAG (2012))
bottom ash treatment, recovery efficiencies for metals	all values		Boesch et al. (2013)
yield of ferrous metal recycling from MSWI	72	%	Haupt et al. (2016)
yield of non-ferrous metal recycling	70	%	Allegrini et al. (2015)

2. Description of officially communicated recycling rates

For some materials, the officially communicated recycling rates are collection rates (CR), while for other materials, the intermediate RR (iRR) are published (neglecting the efficiency in the recycling process). Table S1-3 shortly describes the models used to calculate the official-RR, as the sources are only available in German, and indicates which rates are published by the Federal Office of the Environment (FOEN).

Table S1-3: Definition of official recycling rates communicated by the Federal Office of the Environment (FOEN). CR: collection rate, iRR: intermediate recycling rate, RR: recycling rate.

material	rate published	comments	source
PET	iRR	Residues are deducted from collected amount based on assumptions (no measurements of residues), but losses in recycling process and PE are not assessed separately.	FOEN (2013a)
aluminum	iRR	Based on the anticipated recycling fee, the consumption of aluminum beverage cans is assessed (including the consumption of imported beverages, excluded are exported cans). From recyclers and traders, the collected amount is sourced. The amount of aluminum cans and packaging in the mixed metal collection is assessed annually. The official-RR only includes used beverage cans (UBC). If residues are assessed is unknown, assumptions on residues are available.	FOEN (2013b)
tinplate	iRR	Recycling rate is not assessed, but based on a survey.	Personal communication with Ferro Recycling AG (October 2015)
paper and cardboard	CR	The recycling rate is based on the consumption of paper in the specific year which is based on production data of the companies and import statistics. The amount of recycled paper and cardboard is assessed at the recycling companies (exports of waste paper are deducted).	RPK (2015)
glass	CR	Only the consumption of glass bottles (on which there is an anticipated recycling fee) is assessed. The share of glass from glass bottles in the collection is then estimated (75-78%) for the calculation of the recycling rate. Impurities are not deducted and losses in the recycling are not taken into account.	FOEN (2013c)

3. Data sources for in-depth material flow analyses

3.1 PET bottles

Data used for the material analysis of PET is mainly sourced from the annual report of the association PET-Recycling Switzerland or from internal statistics (unpublished). In addition, the Swiss Federal Customs Administration (Swiss-Impex) is used as a data source for imports and exports.

Table S1-4: Data source of material flow analysis (MFA) of PET in MSW. Reference year is 2012. PRS: PET-Recycling Switzerland (association), MSWI: municipal solid waste incineration, FOEN: Federal Office of the Environment

flow	value	unit	source
export, from collection	700	t	personal communication PET-Recycling Switzerland (October 2015): internal statistics (unpublished)
PET collection non-PRS members	2,440	t	personal communication PET-Recycling Switzerland (October 2015) internal statistics (unpublished)
PET collection PRS members	44,532	t	personal communication PET-Recycling Switzerland (October 2015) internal statistics (unpublished)
other exports	1,585	t	Swiss-Impex (2015), customs tariff number 3915.9000.911
non-recyclables from collection PRS members	6,800	t	annual report of PET-Recycling Switzerland (PRS 2012)
non-recyclables from collection others	386	t	Same share of residues assumed as found in collection of PRS members.
non-recyclables to MSWI	42	%	personal communication PET-Recycling Switzerland (October 2015): internal statistics (unpublished)
non-recyclables to cement kiln	58	%	personal communication PET-Recycling Switzerland (October 2015): internal statistics (unpublished)
export share of PET from PRS members	5.3	%	annual report of PET-Recycling Switzerland (PRS 2012): internal statistics (unpublished)
share of open-loop for exported PET and non-PRS members	100	%	personal communication PET-Recycling Switzerland (October 2015)
share of blue/colorless PET	63	%	annual report of PET-Recycling Switzerland (PRS 2012)
share of colored (green, brown) PET	37	%	annual report of PET-Recycling Switzerland (PRS 2012)
closed-loop recycling: yield closed-loop material	68	%	Kleger (2014), verified by RecyPET AG, Switzerland
closed-loop recycling: yield open-loop material	19	%	Kleger (2014), verified by RecyPET AG, Switzerland
closed-loop recycling: PE	7	%	Kleger (2014), verified by RecyPET AG, Switzerland
closed-loop recycling: metals	1.5	%	Kleger (2014), verified by RecyPET AG, Switzerland
closed-loop recycling: residues to cement kiln	4.5	%	Kleger (2014), verified by RecyPET AG, Switzerland
open-loop recycling: yield open-loop material	88	%	Kleger (2014), verified by RecyPET AG, Switzerland (assumed also for processes outside Switzerland)
open-loop recycling: PE	6	%	Kleger (2014), verified by RecyPET AG, Switzerland (assumed also for processes outside Switzerland)
open-loop recycling: metals	1	%	Kleger (2014), verified by RecyPET AG, Switzerland (assumed also for processes outside Switzerland)
open-loop recycling: residues to cement kiln	5	%	Kleger (2014), verified by RecyPET AG, Switzerland (assumed also for processes outside Switzerland)
PET consumption	46,341	t	annual consumption of PET (FOEN 2013d)
PET from private consumption	1,890	t	assumption based on Wullschleger (2015)
PET in MSWI	8,963	t	calculated from MFA: amount consumed in Switzerland + amount imported from private shopping trips – material collected for recycling; plausibility check: amounts measured in mixed MSW (7000-8000 t, Steiger (2014)) and littering (not measured, assumption of 15% of PET bottles (1400 t) based on Heeb and Hoffelner (2004) and experts)
losses PE recycling	0	%	assumption by authors (sorting of residues already in PET recycling process)

3.2 Aluminum and tinplate

For the MFA of aluminum and tinplate, the closely interlinked associations IGORA and Ferro Recycling, respectively, were the main data providers. Data was mostly sourced from internal financial reports which are used to collect and redistribute the anticipated recycling fees and are therefore given high credibility. For aluminum, the mass flows are calculated separately for mixed aluminum and used beverage cans (UBC). Flows of tinplate and aluminum to the municipal solid waste incineration are highly uncertain as amounts from households are not assessed.

Table S1-5: Data source of material flow analysis (MFA) of aluminum and tinplate in MSW. FOEN: Federal Office of the Environment, MSWI: municipal solid waste incineration, UBC: used beverage cans.

flow	value	unit	source
aluminum in mixed metal collection	32	%	annual survey of metal collection from households of Carbotech AG (unpublished)
tinplate in mixed metal collection	63	%	annual survey of metal collection from households of Carbotech AG (unpublished)
residues in mixed metal collection	5	%	annual survey of metal collection from households of Carbotech AG (unpublished)
UBC, separately collected	5,951	t	personal communication IGORA (October 2015); data sourced from internal financial reports
metal collection from households to traders	3,891	t	personal communication Ferro Recycling / IGORA (October 2015); calculated from data sourced from internal financial reports
metal collection from households to recyclers	11,484	t	personal communication Ferro Recycling / IGORA (October 2015); data sourced from internal financial reports, UBC deducted
metal collection with direct export	2,474	t	personal communication Ferro Recycling / IGORA (October 2015); data sourced from internal financial reports
mixed metals sent to detinning from traders	2,513	t	personal communication Ferro Recycling / IGORA (October 2015); data sourced from internal financial reports
tinplate sent to detinning from recyclers	5,241	t	personal communication Ferro Recycling (October 2015); data sourced from internal financial reports
tinplate in detinning	6,842	t	personal communication Ferro Recycling (October 2015); data sourced from internal financial reports
detinning, tin recovered	0.22	%	personal communication with Elektrozinn AG
efficiency of steel production	88	%	Remus et al. (2013) (for domestically recycled and exported ferrous scrap); for ferrous scrap from MSWI, an efficiency of 72% is used based on Haupt et al. (2016)
residue treatment (for all residues)	MSWI		assumption by authors, approved by experts
tinplate in MSWI	2,084	t	FOEN (2013d) (tested also with 9,000 t of tinplate in MSWI based on Steiger (2014)).
aluminum in MSWI	2,000	t	Assumption. Based on 787 t UBC (recycling rate from (FOEN 2013e)) and 1,300 t aluminum packaging with a assumed recycling rate of 66%.

3.3 Paper and cardboard

The MFA of paper and cardboard was set up in close collaboration with the respective companies as well as the association recycling of paper and cardboard (Recycling Papier und Karton, RPK). Table S1-6 contains the values shown in the MFA (figure 5 in the manuscript) in black. In addition, primary data used for the calculation of values used in the MFA is shown in grey.

Table S1-6: Data source of material flow analysis (MFA) of paper and cardboard in MSW. MSWI: municipal solid waste incineration. Rows in grey are not used for the MFA directly but instead in the calculation of the input values of the MFA.

flow	value	unit	source
paper collected	640,943	t	calculated: 755,884-162,245-0.81*149,394+156,032+5,700+6581<br>from paper in paper recycling incl. residues and paper sent to cardboard recycling or insulation production (see explanations in the following rows)
paper treated in paper recycling	755,884	t	personal communication with association Recycling Paper und Karton (internal accounting document))
waste paper imported	162,245	t	Swiss-Impex (2015), customs tariff number 4707.2 and 4707.3
imported mixed paper	149,394	t	Swiss-Impex (2015), customs tariff number 4707.9; 19% cardboard, 81% paper (as assumed for Switzerland based on industrial experts)
used paper export	156,032	t	Swiss-Impex (2015), customs tariff number 4707.2 and 4707.3
paper to insulation production	5,700	t	personal communication with Isofloc AG
cardboard sorted out at paper recycling plant	6,581	t	personal communication with Utzenstorf AG (November 2015)
cardboard collected	364,912	t	calculated: 362,683-80,826-13,317-0.19*149,394+131,038+300-6,581<br>(see explanation in the following rows)
cardboard treated in cardboard recycling	362,683	t	personal communication with association Recycling Paper und Karton (internal accounting document))
mixed paper collected and not sorted	80,826	t	personal communication with association Recycling Paper und Karton (internal accounting document)
waste cardboard imported	13,317	t	Swiss-Impex (2015), customs tariff number 4707.1
export of used cardboard	131,038	t	Swiss-Impex (2015), customs tariff number 4707.1
cardboard used for the production of insulation material	300	t	personal communication with Isofloc AG
composition of mixed paper	19 / 81	%	mixed paper is assumed to consist of 19% cardboard and 81% paper (by mass, Chappuis et al. (2014))
export of mixed paper	252,154	t	Swiss-Impex (2015), customs tariff number 4707.9
modelling of paper recycling	all transfer coefficients		personal communication with Utzenstorf AG and Perlen AG and Elbert (2014)
modelling of cardboard recycling	all transfer coefficients		personal communication with Model Group AG and Elbert (2014)
residues in cardboard recycling	8.2	%	personal communication with association Recycling Paper und Karton (internal accounting document)
cardboard in MSWI	63,000	t	Steiger (2014)
paper in MSWI	320,271	t	Steiger (2014): 83,000 t of paper and 137,000 t of toilet tissue in MSWI;<br>Hirschberger (2009): 100,271 t of toilet tissue in sewage sludge (ultimately in MSWI)
processes for exported materials			assumption: equal transfer coefficients as in Switzerland

3.4 Glass

Data for the MFA of glass was mostly sourced from the annual report of VetroSwiss and the national statistics by the Federal Office of the Environment. In addition, Meylan (2013) offered an overview of the system and additional statistics.

Table S1-7: Data source of material flow analysis (MFA) of packaging glass in MSW. Reference year is 2012. FOEN: Federal Office of the Environment, MSWI: municipal solid waste incineration

flow	value	unit	source
amount of glass separately collection	353,809	t	annual statistics FOEN (FOEN 2013e)
residues in collection	4.5	%	FOEN (2013c)
residues in closed-loop recycling additionally	4.5	%	assumed by author, approved by industrial experts
reuse of bottles	1.5	%	annual report of VetroSwiss (VetroSwiss 2012)
share of mixed color collection	28.73	%	annual report of VetroSwiss (VetroSwiss 2012)
modeling of closed-loop recycling			annual report of VetroSwiss (VetroSwiss 2012)
share of separate color collection	70.06	%	annual report of VetroSwiss (VetroSwiss 2012)
green glass in color separate collection	60	%	presentation of VetroSwiss (Stuker 2012)
white glass in color separate collection	30	%	presentation of VetroSwiss (Stuker 2012)
brown glass in color separate collection	10	%	presentation of VetroSwiss (Stuker 2012)
share of glass used in foam glass production	9	%	annual report of VetroSwiss (VetroSwiss 2012)
residues in foam glass production	0	%	assumption, approved by experts
share of glass used in packaging glass production	25.35	%	annual report of VetroSwiss (VetroSwiss 2012)
share of exported glass	65.63	%	annual report of VetroSwiss (VetroSwiss 2012)
share in packaging glass production, green	80	%	Meylan (2013)
share in packaging glass production, brown	10	%	Meylan (2013)
share in packaging glass production, white	10	%	Meylan (2013)
glass in municipal solid waste incineration	60,000	t	Steiger (2014)

References

Allegrini, E., A. Boldrin, and T.F. Astrup. 2015. Life cycle assessment of metal recovery from municipal solid waste incineration bottom ash. *Journal of Environmental Management* 151: 132–143.

Batrec Industrie AG. 2012. *Massen- und Energiebilanz 2011 [material and energy balance 2011]*. Wimmis, Switzerland.

Boesch, M., C. Vadenbo, and D. Saner. 2013. An LCA model for waste incineration enhanced with new technologies for metal recovery and application to the case of Switzerland - Supplementary Information. *Waste Management*.

Chappuis, V., A. Probst, and M. Schneeberger. 2014. Ausbau Bereich Mischpapier bei der Altpapierwerk Utzenstorf AG [Extension of mixed paper treatment in paper recycling at Utzenstorf AG]. CAS certification thesis. Faculty of Business, Economics and Social Sciences. University of Bern, Switzerland.

Dettli, R., R. Fasko, U. Frei, and F. Habermacher. 2014. *Transformation der Abfallverwertung in der Schweiz für eine hohe und zeitlich optimierte Energieausnutzung [Transformation of waste management in Switzerland towards a high and time-wise optimal energy recovery]*. Zurich, Switzerland. http://www.econcept.ch/uploads/media/1407_6062_01_Schlussbericht_def_neues_Titelblatt.pdf.

Elbert, F. 2014. Materialflussanalyse des Papier- und Kartonkreislaufs der Schweiz [Material flow analysis of the paper and cardboard cycles in Switzerland]. Bachelor thesis. Institute of Environmental Engineering. ETH Zurich. Zurich, Switzerland.

FOEN (Swiss Federal Office of the Environment). 2013a. *Faktenblatt: Berechnung der Verwertungsquote von PET-Flaschen [Factsheet: Calculation of recovery rate of PET bottles]*. Federal Office of the Evironment. Bern, Switzerland.

FOEN (Swiss Federal Office of the Environment). 2013b. *Faktenblatt Berechnung der Verwertungsquote von Alu-Dosen [Factsheet: Calculation of recovery rate of aluminum beverage cans]*. Federal Office of the Evironment. Bern, Switzerland.

FOEN (Swiss Federal Office of the Environment). 2013c. *Faktenblatt Berechnung der Verwertungsquote von Altglas-Flaschen [Fact sheet for the calculation of recycling rate of post-consumer glass bottles]*. Federal Office of the Evironment. Bern, Switzerland.

FOEN (Swiss Federal Office of the Environment). 2013d. *Recycling von Getränkeverpackungen 2012 [recycling of beverage containers 2012]*. Federal Office of the Environment. Bern, Switzerland.

FOEN (Swiss Federal Office of the Environment). 2013e. *Abfallmengen und Recycling 2012 im Überblick [Overview of waste amounts and recycling in 2012]*. Federal Office of the Environment. Bern, Switzerland.

Guerra, F. and B. Kast. 2015. *Bauabfälle in der Schweiz - Hochbau Studie 2015*. For: Federal Office of the Environment. Zurich, Switzerland.

Haupt, M., C. Vadenbo, C. Zeltner, and S. Hellweg. 2016. *Influence of input-scrap quality on the environmental impact of secondary steel production*. *Journal of Industrial Ecology*. DOI: 10.1111/jiec.12439 [online].

Heeb, J. and W. Hoffelner. 2004. *Litteringstudie - Zwischenbericht [Littering study - intermediate report]*.

Hirschberger, P. 2009. *Hygienepapier-Konsum und die Schweiz [Consumption of toilet tissue in Switzerland]*.

KEBAG (Kehrichtbeseitigungs-AG). 2012. *Jahresbericht 2012 [annual report 2012]*.

Kleger, P. 2014. Massenflussanalyse von PET-Recycling in der Schweiz [Material flow analysis of PET-recycling in Switzerland]. Bachelor thesis. Institute of Environmental Engineering. ETH Zurich. Zurich, Switzerland.

Meier, D. 2015. Materialflussanalyse und Ökobilanz von Textilrecycling in der Schweiz [material flow analyis and life cycle assessment of Swiss textile recycling]. Bachelor thesis. Institute of Environmental Engineering. ETH Zurich. Zurich, Switzerland.

Meylan, G. 2013. Integrating stakeholder perspectives into policy support of municipal solid waste management. Diss. ETH Nr. 21495. Zurich, ETH Zurich.

PRS (PET Recycling Schweiz). 2012. Verein PRS PET-Recycling Schweiz. Geschäftsbericht 2012. [Association PRS PET-Recycling Switzerland. Annual report 2012].

Remus, R., S. Roudier, M. a. Aguado Monsonet, and L.D. Sancho. 2013. *Best Available Techniques (BAT) Reference document for iron and steel production*. EUR 25521 EN. European Commission. Seville, Spain.

RPK (Recycling Papier und Karton). 2015. *Altpapiergesamtstatistik 2015 [post-consumer statistics 2015]*. Association Recycling Papier und Karton. Bern, Switzerland.

Schelker, R. and P. Geisselhardt. 2011. *Projekt „ Kunststoff-Verwertung Schweiz “*.

Schneider, M. and S. Rubli. 2007. *Ressourcenmodell mineralischer Baustoffe auf der Ebene Stadt Zürich [Ressource model for mineral construction materials in the city of Zurich]*.

Silva, N. da, N. D'Souza, and M. Binder. 2010. *Life Cycle Impact Assessment of Aluminum Beverage Cans*. PE Americas, Boston, United States of America.

Steiger, U. 2014. *Erhebung der Kehrichtzusammensetzung 2012 [Assessment of waste composition 2012]*. Federal Office of the Environment (FOEN). Bern, Switzerland. http://www.news.admin.ch/NSBSubscriber/message/attachments/33597.pdf.

Stuker, F. 2012. *Vorgezogene Entsorgungsgebühr für Glasflaschen*.

Swiss-Impex. 2015. Swiss Federal Customs Administration. https://www.swiss-impex.admin.ch/. Accessed last October 2015.

Tschümperlin, L., S. Büsser, and R. Frischknecht. 2015. *LCI of plastic recovery options Authors*. Internal project report (unpublished). treeze GmbH, Uster, Switzerland.

VetroSwiss. 2012. *Jahresbericht 2012 [annual report 2012]*.

Wäger, P.A., R. Hischier, and M. Eugster. 2011. Environmental impacts of the Swiss collection and recovery systems for Waste Electrical and Electronic Equipment (WEEE): a follow-up. *The Science of the Total Environment* 409(10): 1746–56.

Wullschleger, S. 2015. Bilanzierung von Abfällen aus privaten Auslandseinkäufen [Accounting of waste from private shopping abroad]. Bachelor Thesis. Institute of Environmental Engineering. ETH Zurich.
